# Applying the AOGCM-AR5 models to the assessments of land suitability for walnut cultivation in response to climate change: A case study of Iran

**DOI:** 10.1371/journal.pone.0218725

**Published:** 2019-06-27

**Authors:** Kourosh Vahdati, Ali Reza Massah Bavani, Morteza Khosh-Khui, Poya Fakour, Saadat Sarikhani

**Affiliations:** 1 Department of Horticulture, College of Aburaihan, University of Tehran, Tehran, Iran; 2 Department of Irrigation and Drainage, College of Aburaihan, University of Tehran, Tehran, Iran; 3 Department of Horticultural Science, College of Agriculture, Shiraz University, Shiraz, Iran; Potsdam Institute for Climate Impact Research, GERMANY

## Abstract

Due to higher temperatures and lower water availability, climate change is likely to have a major impact on walnut production in the near future. Climate change will alter the land suitability for walnut cultivation around the world, especially in arid and semi-arid regions like Iran. Here, land suitability for the cultivation of walnut (*Juglans regia* L.) in Iran was determined using the GIS for present and future conditions (2020–2049) with an approach to climate change. Accordingly, data from 375 synoptic stations throughout Iran were gathered for climatic factors including average, minimum and maximum temperatures, relative humidity and chilling requirement. Also, ASTER sensors (Advanced Spaceborne Thermal Emission and Reflection Radiometer) and their data provided this research with cells that make a precision of 150 m (5 s), and the data were used for gauging geological parameters such as altitude and land slope. The electrical conductivity (EC) of soil and water were informed by the data bank of the Iranian Water Resources Management. The results of temperature simulations for the future (2020–2049) were analyzed by 21 AOGCM-AR5 models under the RCP4.5 emission scenario. In the first phase of evaluations, the maps of land suitability were constructed for present conditions by considering a network of the above-mentioned parameters. By combining these layers of information, the final map of land suitability was illustrated for walnut cultivation. In the second phase, the NEX-GDDP was used in order to determine land suitability for the future (2020–2049). The results showed that Iran currently has 582844 km^2^ of land suitable for walnut cultivation. However, the future will see less suitable lands: the current area will be reduced by 6.19%, from 582844 km^2^ to 546710 km^2^. In general, the northern, northwestern and western margins of Iran are currently suitable for walnut cultivation. By approximation, these lands will also be major areas for prospective cultivations of walnut in the future (2020–2049), even though their current stretch will be reduced.

## Introduction

Iran is a vast country in the Middle East. It has a variety of climates because of its two mountain ranges (Alborz and Zagros). The country is further endowed with long coastlines in its south and north [1]. Also, several other mountains in Baluchestan, Khorasan and the central parts of Iran provide a variety of microclimates [2]. Although the main climate of Iran is extremely dry and semi-arid, its north is characterized by a wet climate. The west of Iran is also considered as relatively wet. Apart from the south of the Caspian Sea, where the annual preciption is almost high and evenly distributed throughout the year, most parts of Iran have moderate to low rainfall which occur mostly from October through April [1]. Agricultural land suitability in Iran is very limited due to multiple limiting factors including high sodium contents in the soil, high EC of the soil and water, low precipitation, low amounts of organic carbon in the soil and steep slopes. In other words, a large part of Iran’s land (around 78.2%) is regarded as unsuitable or poorly suitable areas [3]. Despite these limitations, Iran has experienced the cultivation of many different horticultural crops, especially nut crops, because of the country’s diverse climates. In fact, Iran is one of the main centers of origin for many temperate fruit trees, including walnuts [4, 5].

Nut crops are one of the most important horticultural crops in the world. Their production has increased by more than 24% (equivalent to 2.4 million metric tons of nut kernel) through the past ten years. In particular, almonds and walnuts have had the highest growth rates in the world, among other nut crops [6]. Persian walnuts (*Juglans regia* L.) are widely cultivated across the temperate regions of the northern hemisphere, especially from 30° to 50° (in Asia, Europe and North America) as well as in the southern hemisphere from 30° to 40° (in Australia, New Zealand, South Africa, Chile and Argentina) [7]. The Persian walnut originated in ancient Persia and spread to its western neighbors and central Asia [2]. Iran is the fourth leading nut crop producer and its total production accounts for 7% of the world’s total production. The country produces 79% and 13% of the pistachios and walnuts in the world, respectively [6]. After the pistachio, walnut is the most important nut crop in Iran, and there have been growing demands for its production and consumption in recent years [2]. Iran is one of the main centers of origin for walnuts and ranks third as a leading producer of Persian walnuts in the world (with around 349192 tons) after China and the United States. Walnut production and its area of harvest in Iran have increased by 63% and 58.9%, respectively, through the last 10 years [8].

Land suitability evaluation is to estimate the inherent capacity of any unit of land that could support specific practices and uses for a long period without deterioration [9, 10]. The evaluation of land suitability not only indicates the type of land use but also shows its limitations in the production of a given type of crop [11]. Geographic information systems (GIS) and remote sensing data are commonly used for land suitability evaluation. GIS has been used as a tool for developing alternative uses of agricultural land, precision farming and land suitability mapping [12, 13].

In previous studies, the GIS-based approach has been used in order to evaluate land suitability for various crops in different areas [9, 14, 15, 16, 17, 18]. Mesgaran et al. [3] evaluated Iran’s land for agriculture using GIS information. They reported that only 2.4% of Iran’s land is either suitable or highly suitable for agricultural crop production. Also, the majority of highly suitable lands for agriculture are located in the northern and western provinces of Iran [3]. The GIS approach has assisted the evaluation of land suitability for apple and pear cultivation in the Uttarankhand region, north of India, and it was reported that the south of Uttarankhand is more suitable for pome fruit production. In contrast, its upper hilly region was found to be unsuitable for the cultivation of apples and pears due to the presence of clay soils and chilling injuries [9]. The evaluation of land suitability for walnut cultivation in Beijing, China, involved the use of GIS data and showed that low mountainous regions situated in the west and north of Beijing are suitable for walnut cultivation [15].

Over the past recent decades, climate change has significantly affected agricultural activities on a global scale. The consequences of climate change on temperate-zone fruit trees include drought stress, earlier flowering among trees, possible risks of more damage due to late-spring frost, the decline in the number of chill hours in the winter and deprivation of the chilling requirement [19, 20]. According to the Intergovernmental Panel on Climate Change (IPCC), most countries will experience an increase in their average temperatures, more frequent heat waves, scarcer water resources, desertification, and irregular periods of heavy precipitation [21]. The consideration of climate change and its effects on land suitability has become an important issue concerning food security [22]. In this context, Zhao and Lin [23] reported that climate change would reduce the area of suitable lands for *Jatropha* cultivation in the world. It was suggested that the predicted scenarios of climate change would reduce the suitability of *Jatropha* cultivation in areas near the equator [23].

After pistachio, walnut is the most important nut crop in Iran. In recent years, the country has experienced a rise in demand for newly established, modern walnut orchards by fruit growers. Due to the effects of climate change on suitable lands for walnut cultivation, this study was conducted to determine land suitability for walnut cultivation using GIS. Two temporal spans were considered in this research: the present-day conditions of walnut cultivation and the future of cultivation (2020–2049) as affected by simulations of climate change and the speculations thereof.

## Material and methods

**Fig 1:**
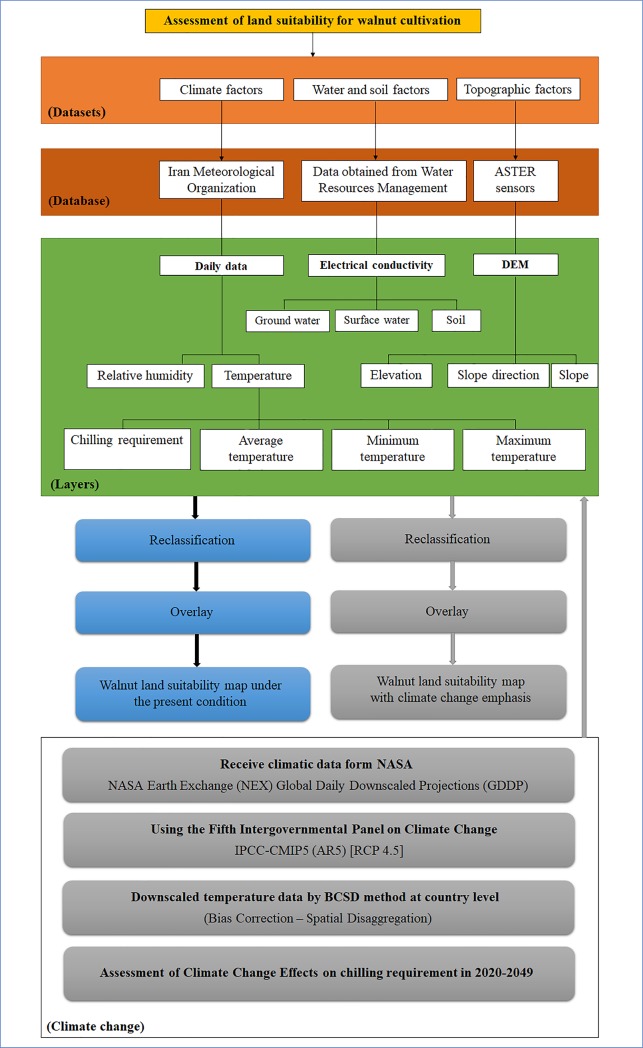
Conceptual model to prepare the maps of land suitability for walnut cultivation in Iran using GIS.

In this study, we evaluated the entirety of Iranian territory for walnut production using GIS information. Iran has a total area of 1,648,195 km^2^ and is the world’s 17^th^ largest country [24]. This study was performed in two phases. In the first phase, land suitability for walnut cultivation in Iran was determined for the present conditions. In the next phase, the effects of climate change were estimated for the next thirty years (2020–2049) in the areas of study. Fig 1 shows the conceptual model used for this study.

### Land suitability for walnut cultivation in Iran under the current conditions

In the first phase, the climatic (maximum, average and minimum temperature, relative humidity and chilling requirement), edaphic (water and soil EC) and topographic (altitude and land slope) data of walnut production were used. The climate data obtained from the Iranian Meteorological Organization (375 synopetic stations). The climatic data included ambient temperatures in the growing season, minimum winter temperatures and the maximum temperature during the year, chilling requirement and relative humidity. The calculations were performed using long-term statistics (spanning from 30 to 67 years) among 375 synoptic stations in Iran. The ArcGIS software was used for analyzing the data. The climatic data from synoptic stations were interpolated by applying the Kriging method of prediction maps in the form of layers.

In order to classify the chilling requirements in all regions of Iran, along with the consideration of daily winter temperatures (December, January, and February), the raw data were converted to hourly data using the Monteith and Unsworth [25] method according to the following formula:

$$\mathrm{Hourly}\;\mathrm{temperature}=\frac{\left(T\max+Tmin\right)}{2}+\frac{\left(Tmax-Tmin\right)}{2}\times \sin(radian\;(H\times 15)+210)$$

Tmax: Daily maximum temperature

Tmin: Daily minimum Temperature

H: Hour of day

The ASTER (Advanced Spaceborne Thermal Emission and Reflection Radiometer) sensors provided information regarding altitude and land slopes. In this study, a digital elevation model (DEM) was used which had a precision of 150 m (5 seconds). Usually, a digital elevation model (DEM) is used in most spatial GIS analysis and can be used for mapping terrestrial slopes and their directions. It can assist various analyses in a three-dimensional fashion.

The edaphic data in this study were prepared based on a database from the Iran Water Resources Management Company (http://www.wrm.ir/). To analyze the edaphic data, the method of interpolation was used based on Kriging method by using ArcGIS software. The map was predicted with regard to the electrical conductivity (EC) of the soil, and the EC of surface and underground waters.

Iran’s land was classified into four suitability class based on the climate, edaphic and topography variables which is important for walnut production (Table 1). These suitability classes were named as highly suitable, suitable, fairly suitable and unsuitable. This classification was conducted based on the literature review [7, 26, 27] and the comments of expert walnut researchers in the world who advise on commercial walnut production. Highly suitable pedoclimate conditions are considered as ideal conditions for planting walnuts. Suitable conditions are lands which share a proximity with the ideal conditions for the planting. Fairly suitable conditions are those in which the cultivation of walnuts is possible but with some restrictions. Unsuitable conditions apply to conditions where the success of planting walnuts is not technically and economically feasible.

**Table 1:** Classification of pedoclimate condition of walnut for land suitability assessment.

Pedoclimate Condition	Highly Suitable (Best)	Suitable	Fairly Suitable	Unsuitable
**Altitude (m)**	0–1000	1000–2000	2000–3000	>3000 and <0
**Average Temperature in Growth Season (°C)**	20–28	18–20	15–18	<15
**Min. Temperature in Winter (°C)**	-5<	-5>Suitable>-15	-15>Fairly Suitable>-20	< -20
**Max. Temperature (°C)**	30>	30–35	35–38	>38
**Chilling Requirement (0–7°C)**	1100–1800	900–1100	600–900	<600
**Soil EC (ds/m)**	1.5>	1.5–2.5	2.5–4	4<
**Surface Water EC (ds/m)**	1.5>	1.5–2.5	2.5–4	4<
**Groundwater EC (ds/m)**	1.5>	1.5–2.5	2.5–4	4<
**Relative Humidity in Summer (%)**	45–65	30–45	20–30	20> and 65<
**Land Slope (%)**	0–5	5–15	15–30	30<

The base maps provided from free shapefile maps which obtained from Iran Water Resource Management Company. The fuzzy algebraic product model was applied to combine the layers and determine land suitability for walnut cultivation. Here, the membership of an element in a set is defined with a value in the range of one (full membership) to zero (non-full membership). The degree of membership is usually expressed by a membership function whereby the form of the function can be linear, nonlinear, continuous or discontinuous. In a fuzzy model, each pixel in each map is assigned a value between zero and one which indicates the amount. The location of the pixel is inappropriate from the point of view of the relevant criterion for the target [28].

### Land suitability for walnut cultivation in Iran for the future (2029–2049)

In the second phase, the NASA Earth Exchange Global Daily Downscaled Projections (NEX-GDDP) dataset was used, and land suitability for walnut cultivation was determined by considering an approach to climate change during the years 2020–2049. The NEX-GDDP climatic data were obtained from https://cds.nccs.nasa.gov/nex-gddp and the ensemble of 21 AOGCM models was used by equally weighting them. For this purpose, the BCSD downscaling method was originally designed to process monthly data. This algorithm assumes that the biases in the current and future simulations follow the same pattern. To perform the BCSD algorithm, three databases are required: observational data, AOGCM model data for the base period and AOGCM model data for future periods. The bias correction is applied to the first and second databases. At first, the observation data and the base period of the AOGCM models are converted to the resolution (e.g. 0.1°×0.1°). Then, in each cell, the cumulative probability distribution function (CDF) of observation data and the base period of the AOGCM models are calculated. The relationship between these two CDFs has been established to draw a correlation. Finally, this relationship will be used for overriding the future data of the AOGCM model [29]. The parameters in the second phase included the minimum and maximum daily temperatures and the chilling requirement.

Variations among the maximum and minimum temperatures in the 2020–2049 period were determined in comparison with the 1976–2005 baseline period. Firstly, the delta ratio values (ΔT) of the maximum and minimum temperatures between the 30-year average monthly values were calculated for the upcoming period (2020–2049) and then the simulated baseline period (1976–2005) was calculated by the same model for each cell according to the following equation [30]. These values estimate a 30-year average of climate change, as considered in relation to the baseline period. The baseline period refers to data belonging to 1976–2005 which can also be mentioned in the formula as the 'base.'

$$\Delta T_{i}=\left({\bar{T}}_{\mathrm{GCM},\mathrm{fut},i}-{\bar{T}}_{\mathrm{GCM},\mathrm{base},i}\right)$$

**₸ GCM, fut, i:** 30-year average temperature simulated by AOGCM in the future period (2020–2049) for each month

**₸ GCM, base, i:** 30-year average temperature simulated by AOGCM during the baseline period (1976–2005) with observations for each month

In this study, the temperature delta values were calculated for all 21 AOGCM models. Then, the median of values for the 21 models pertaining to each month were calculated. These were set as criteria for determining the country’s climatic changes.

## Results

### Phase I: Determination of land suitability for walnut cultivation under the present conditions

**Fig 2:**
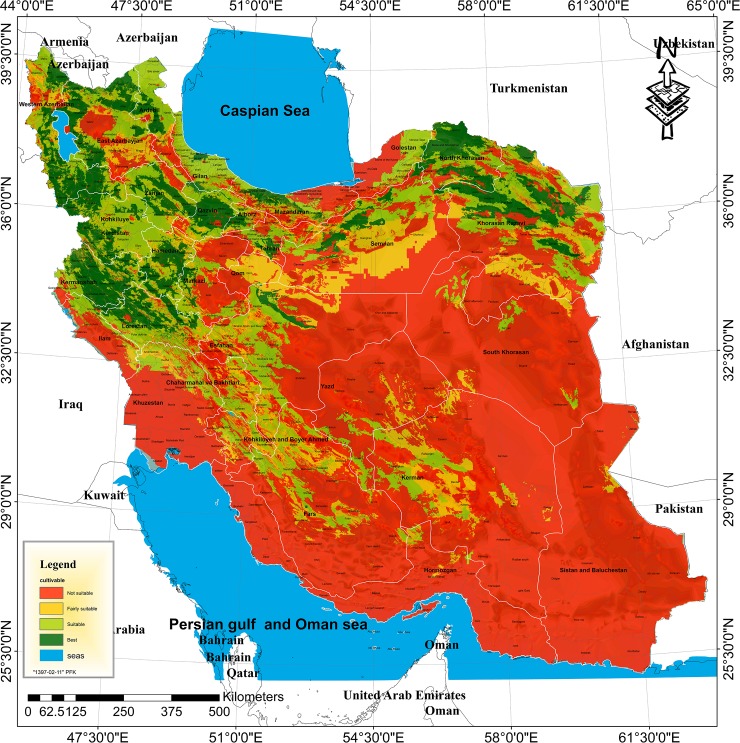
The map of land suitability of walnut cultivation in Iran for current conditions.

The ultimate structure of mapping suitable lands for walnut cultivation in Iran was established here by combining the layers pertaining to the average air temperature in the growing season, the maximum temperature in summer, the minimum temperature in winter, the chilling requirement, the relative humidity of the air during the growing season, the altitude, land slope, EC of the soil, surface water and groundwater (S1 Fig). Fig 2 illustrates the zoning of land suitability for walnut cultivation in Iran. It consists of cells, each of which has dimensions of 150 meters (2.2 ha). The results revealed that the total amount of unsuitable lands for walnut cultivation are 1065351 km^2^ (106535100 ha) which comprises 64.64% of the total area of Iran (Table 2). The majority of unsuitable lands for walnut cultivation were in the south of Iran. These lands comprise parts of the provinces of Khuzestan (except a part of Andimeshk, Dezful, Masjed Soleiman and Izeh cities), Bushehr, Hormozgan (except a part of Haji Abad city), Sistan and Baluchestan and Fars (including Farashband, Mohr, Qir and Karzin, Khonj, Jahrom, Larestan, Zarin Dasht and Darab cities) and Kerman (including Qaleh Ganj, Rodbar-e-Jonubi, Manujan, Kahnuj, Anbarabad, Jiroft, Bam and Kerman cities). In addition to the south of Iran, the unsuitable lands were also mapped in parts of the central regions of Iran such as the south of Semnan and Yazd provinces (including Ardekan, Meybod, Behabad, Ashkezar and Yazd cities) and South Khorasan (except Nehbandan and Sariyan cities). These lands can be considered as unsuitable for walnut cultivation because of a lack of sufficient winter chill hours which are necessary to meet the chilling requirement. The mentioned lands are also characterized by unfavorably high EC values in the soil and in the water resources, along with high summer temperatures (higher than 38°C) (Fig 2).

**Table 2:** Land suitability for walnut cultivation in Iran in the present and future (2020–2049).

Cultivation ability		Present		Future	
		Area (km^2^)	Share of Iran’s area	Area (km^2^)	Share of Iran’s area
	**Highly suitable (best)**	150433	9.13	132525	8.04
	**Suitable**	275213	16.70	293448	17.80
	**Fairly suitable**	157198	9.54	120737	7.33
Cultivable*		582844	35.36	546710	33.17
**No cultivable (Unsuitable)**		1065351	64.64	1101485	66.83

*The total walnut cultivable area are the sum of highly suitable, suitable and fairly suitable.

Based on the results, parts of the Caspian coastline in northern Iran, especially parts of Golestan, Mazandaran and Gilan provinces, have relative humidity levels of more than 65 percent and thus are not recommended for walnut cultivation (Fig 2).

The results revealed that parts of the provinces of Khorasan Razavi (including Meh Valat, Bajestan, Gonabad, Rashtkhar and Khalilabad cities), Qom, Isfahan (including Aranbidgol and Ardestan cities), Semnan (including south of Semnan, Garmsar and Shahrud cities), Yazd (Bafgh city), and Kerman (including Bardsir, Baft and Anar cities) were of the “fairly suitable” class of regions for the cultivation of walnut (Fig 2). In addition to these areas, parts of the northwestern provinces of Iran were also included in this class. Accordingly, over 9.54% of the total area of Iran (i.e. 157198 km^2^) can be considered to be in the “fairly suitable” class of regions for walnut cultivation. (Table 2). Unlike unsuitable lands, fairly suitable lands can be potentially considered suitable for the cultivation of walnut, but certain pedo-climatic restrictions may probably incur additional production costs and thus make low yields per unit area.

The results indicated that 150433 km^2^ (i.e., 9.13% of Iran's total area) and 275213 km^2^ (i.e. 16.70% of Iran’s total area) are of the “highly suitable” and “suitable” classes for walnut cultivation, respectively. In other words, minimal limits exist there for walnut production when considering the studied factors (i.e. soil and water EC, relative humidity, temperature, chilling requirement, altitude and land slope). The provinces in the range of Alborz, Zagros and Kopet Dagh mountains are considered as highly suitable and suitable areas for walnut cultivation. Parts of the Fars province, Kerman and Yazd are also among the highly suitable and suitable classes for the cultivation of walnut. In general, the available potential for walnut cultivation comprises about 35.36% of the country’s total area. The majority of this percentage is located in the northern, north-western and western margins of Iran (Fig 2).

### Phase II: Determination of suitability for walnut cultivation in Iran by taking the approach of climate change

**Fig 3:**
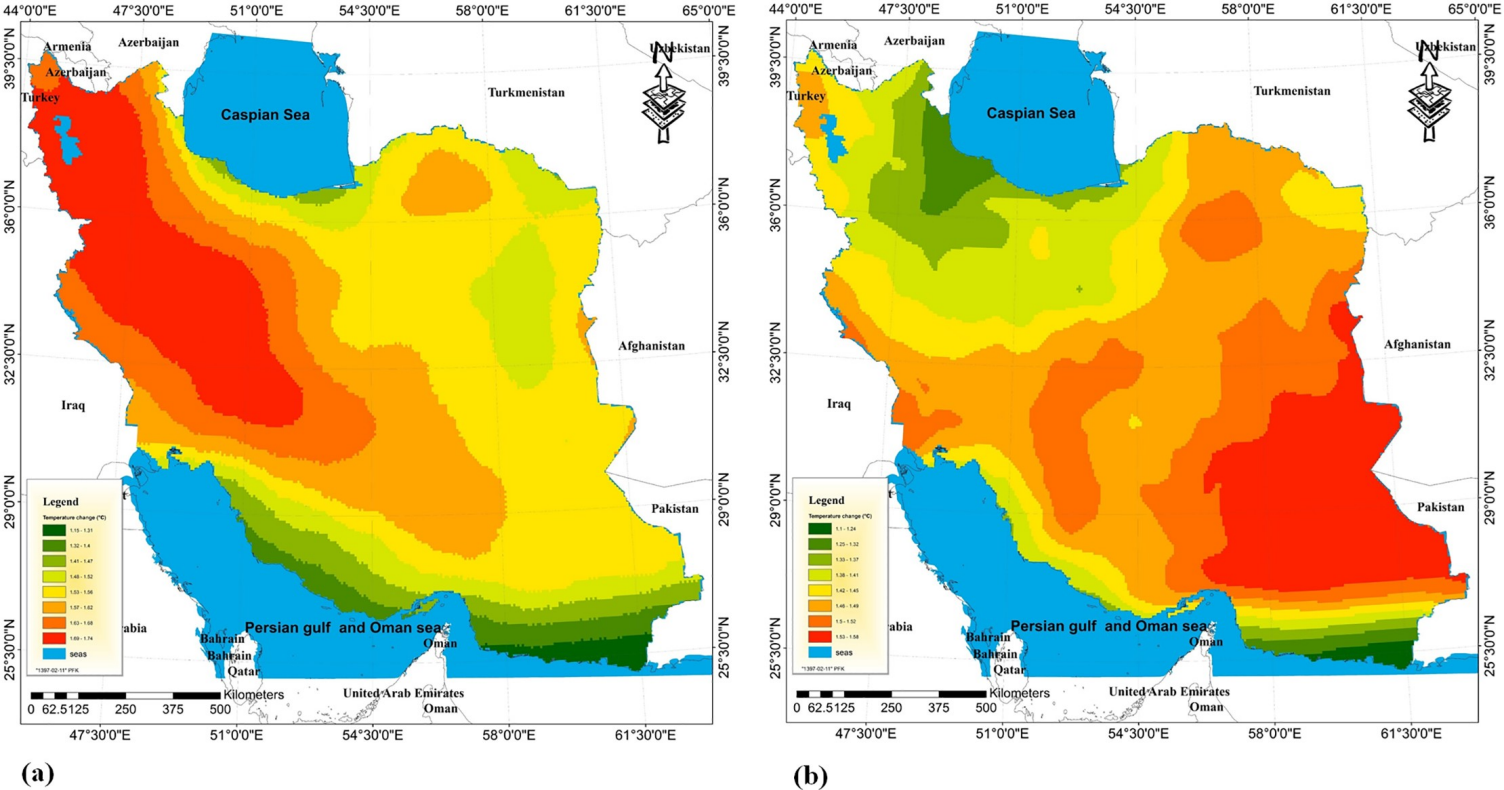
Classification of Iran for walnut cultivation regarding maximum (a) and minimum (b) temperature in the future period (2020–2049).

By simulating the variations in the maximum and minimum temperatures for the future period (from 2020 to 2049), an increase in temperature (around 1.5°C) is indicated throughout all months of the year and in all regions of Iran (Fig 3). This is consistent with the predicted amounts of increase in the fifth report of IPCC (Intergovernmental Panel on Climate Change). The most significant increase in temperature will be observed in the summer months, and the lowest increase is anticipated in the winter and spring. The minimum temperature in the northern half of Iran faces an average increase of 1°C during the winter and spring months. However, the increase will be higher in the southern half of the country and be 1.3°C. In the summer, minimum temperatures are expected to rise substantially in all areas of the country. This increase will be about 1.7°C. Maximum temperatures in the north and north-eastern parts of the country will experience an average increase of 1.53°C. Furthermore, this increase in maximum temperatures will be 1.5 to 1.7°C in December and May, but will be 1.8°C in the summer (S2 Fig). In general, the temperature for all areas of the country will increase by 1.53°C in maximum temperature and 1.45°C in minimum temperature.

Climate change and global warming have substantial effects on the number of chill hours in winter. It is estimated that a significant decrease will occur in the number of chill hours in the coming years [31, 32]. This decline in the chill hours of winter can cause deprivations of the chilling requirement for many fruit trees in temperate regions and could reduce the vastness of the temperate zone [33]. The classification of Iranian regions in terms of the chilling requirement for the future period (2020–2049) showed that the southern provinces of Iran which are close to the Persian Gulf and the Oman Sea (e.g., Sistan and Baluchistan, Hormozgan, and Bushehr) will continue to be unsuitable regions for the cultivation of walnuts for the coming years. This is mostly because of the inability of those regions in providing the chilling requirement of walnut trees.

**Fig 4:**
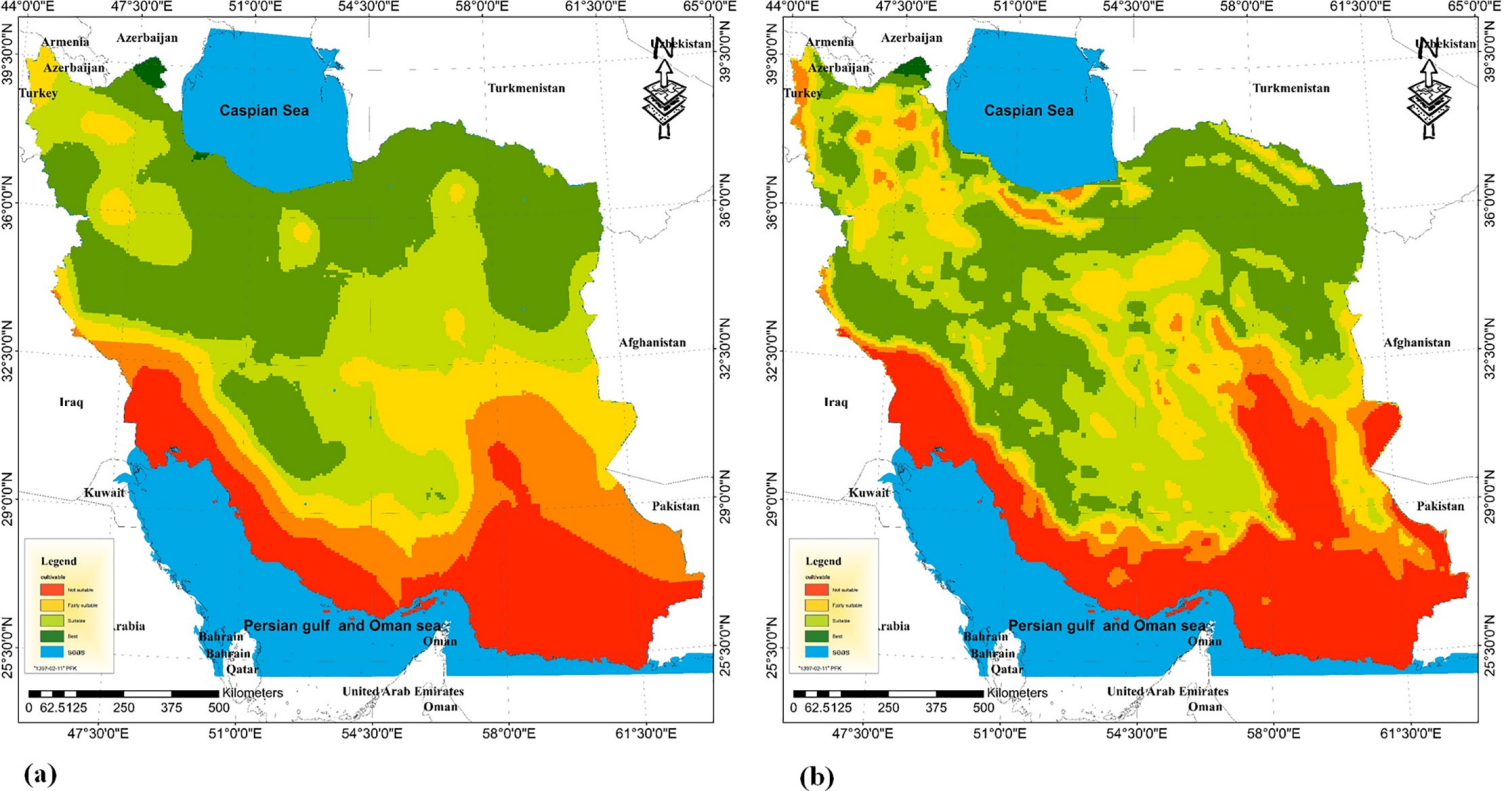
Classification of Iran’s land for walnut cultivation regarding the chilling requirement in the present (a) and future (b).

The potential impact of climate change has been adverse, especially on the horticultural demand for chill hours throughout the country, and the vastness of areas that are currently capable of meeting the chilling requirement will be reduced. In other words, some regions in the ‘highly suitable’ class will be turned into the ‘suitable’ class, and some regions that are currently ‘suitable’ will be turned into the ‘fairly suitable’ class regarding the number of chill hours (Fig 4). On average, there will be 50 hours of decline in the number of chill hours in Iran over the future period. For instance, regions that are currently with a total number of 1600 chill hours will have 1550 chill hours in the future period.

**Fig 5:**
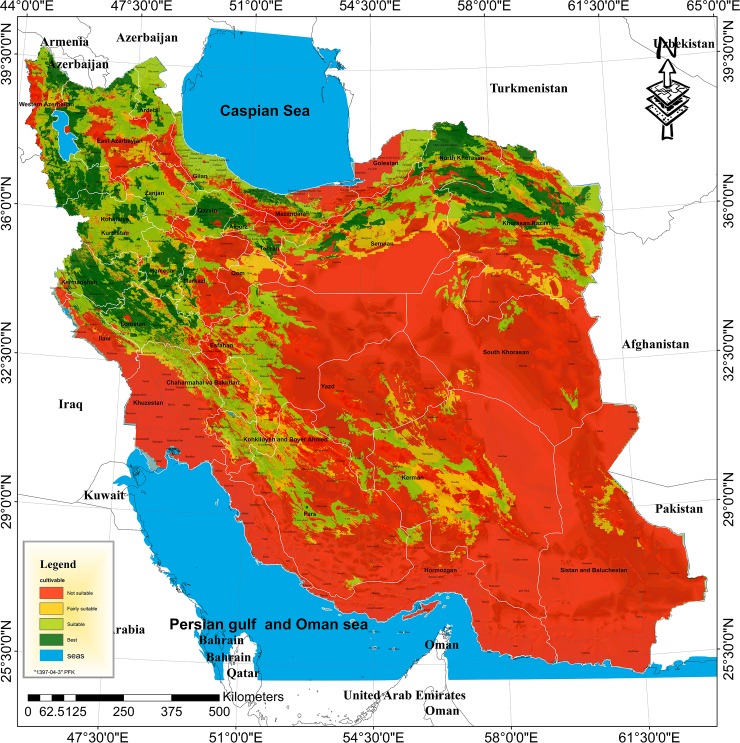
The map of land suitability for walnut cultivation in Iran (2020–2049) with an emphasis on climate change.

Studying land suitability for walnut cultivation in the future of Iran (2020–2049) indicates that the overall appearance of the map overview will be almost identical to the current one. In general, as in the current situation, large parts of the southern and central regions of Iran will remain to be unsuitable for the production of walnuts in the future. In contrast, the northern, northwestern and western margins of Iran (across the Alborz, Zagros and Kopet Dagh Mountains) are currently considered and will continue to be in the “highly suitable” and “suitable” class of regions for the cultivation of walnuts (Fig 5). An exact comparison between the current status (Fig 2) and the future status (Fig 5) of land suitability in Iran shows that many areas that are currently in the “fairly suitable” class of regions for walnut cultivation will be turned into the “unsuitable” class. These include parts of Semnan province, Qom and Isfahan. Also, some “highly suitable” regions in the northwest of Iran (including Zanjan, Ardebil, and East Azarbaijan provinces) and some central regions of Iran (e.g. Isfahan province) will be downgraded to the “suitable” class of cultivation over the future period (Fig 5).

The current conditions suggest that 582844 km^2^ out of 1648195 km^2^ in Iran are cultivable for walnut and are capable of meeting productive expectations. Based on the results, it is estimated that highly suitable lands for walnut cultivation in Iran will be reduced by 11.39% over the next 30 years (2020–2049) due to global warming. This decline in highly suitable lands would be from 150433 km^2^ to 132525 km^2^. This also means that the reduced area of highly suitable lands will be approximately added to the “suitable” class of regions which will increase from 275213 km^2^ to 293448 km^2^. In other words, some of the “highly suitable” class for walnut cultivation in Iran is becoming “suitable” because of climate change. In general, during the next thirty years (2020–2049), climate change will take away 36,136 km^2^ of the country from any prospect for walnut cultivation. Accordingly, 546710 km^2^ of Iran’s land will be suitable for walnut cultivation in the next 30 years. Furthermore, 66.83% of Iran’s land (i.e. 1101485 km^2^) will become unsuitable for walnut cultivation, and only 33.17% (i.e. 546710 km^2^) of the country’s area will be capable of hosting walnuts. The latter percentage is achieved by adding up the highly suitable, suitable and fairly suitable classes (Table 2).

## Discussion

Climate change can adversely affect food security on global and regional scales. It influences the quality and quantity of agricultural products and, generally, the availability of food [34, 35]. Among the consequences of climate change are the increase in temperatures, the decline of rainfall and available water, the erratic fluctuations in rainfall patterns, the changes in flowering patterns of fruit trees, reductions in the number of chill hours and higher risks of late spring frost [19, 20, 21]. Climate change and the rise in urbanization have cut back on agricultural lands, and it is necessary to select suitable areas for the cultivation of crops.

Meanwhile, demands for the establishment of commercial, modern walnut orchards have increased in Iran over the past recent years, and it is a knowledgeable decision to identify suitable areas for walnut cultivation in response to climate change [10]. The results of this study showed that the northern, northwestern and western regions of Iran are highly suitable for walnut cultivation. Among the most important suitable areas for walnut cultivation, we can mention provinces like Khorasan Razavi and North Khorasan, the north of Semnan, the south of Mazandaran, Tehran, Alborz, Qazvin, Zanjan, Gilan, Ardabil, East Azarbaijan, West Azarbaijan, Hamedan, Lorestan, Kurdistan, Kermanshah, Kohgiluyeh and Boyer Ahmad, Chaharmahal and Bakhtiari, in addition to some parts of Fars (including Farashband, Mohr, Qir and Karzin, Khonj, Jahrom, Larestan, Zarin Dasht and Darab cities), Kerman (including Bardsir, Baft and Anar cities), Isfahan (except Ardestan, Isfahan, Nain, Khur and Biabanak cities) and Yazd (including a part of Khatam, Abakuh, Taft, Mehrz and Bafgh cities). Among these provinces, the highest levels of land suitability and cultivation are expected to be in Hamedan, Lorestan, Kurdistan, Kermanshah, Zanjan and West Azarbaijan.

In order to evaluate the validation of the map drawn for land suitability in this study, the results were compared with reports and statistics published by the Iranian Ministry of Agriculture, along with other relevant information and field evaluations [36, 37]. According to a report by the Ministry of Agriculture [36] and Soleimani et al. [37], the production of walnut is mostly centered in the northern and western regions of the country. In the central regions of the country, as reported by the Ministry, only some parts of Isfahan, Kerman, Yazd, and Fars are observed to be under walnut cultivation. Accordingly, the accuracy of the map obtained in this study can be confirmed.

Furthermore, Soleimani et al. [37] published maps of walnut production in Iran, based on statistics of the year 2005 which were provided by the Iranian Ministry of Agriculture (S3 Fig). The researchers reported no instances of walnut cultivation along the Caspian coastline but referred to the populated cultivation of walnuts in the provinces of Kerman, Hamedan, Fars, Lorestan, Kurdistan, and Kermanshah [37]. The map obtained in this study confirms that the mentioned provinces are the most suitable areas for walnut cultivation.

The assessment of land suitability for walnut cultivation in the Tehran province [38] showed that about 50% of the province (i.e. 1,904,307 hectares) is unsuitable for walnut cultivation. The northern parts of the Tehran province (including parts of Shemiranat, Firoozkooh and Damavand) and a southern part of the province (including a part of Varamin city) are unsuitable areas for walnut cultivation. The greatest limits to walnut cultivation in the north and south of the Tehran province are high altitude and high values of EC in the water and soil, respectively. In addition, the occurrence of high temperatures during the growing season is another limiting factor for walnut cultivation in Varamin, Tehran province. These results confirm a previous study on land suitability for walnut cultivation in the Tehran province [38].

The study of climate change and its effects on the land suitability of walnut cultivation in Iran showed that climate change is capable of increasing the temperature by 1–1.7°C during the growing season, and would reduce the number of chill hours in the winter by 50 hours over the next 30 years (2020–2049) [39, 40]. This increase in temperature and the deficiency of chill hours will significantly reduce the suitable areas for walnut cultivation from 582844 km^2^ to 546710 km^2^, and these results confirm previous research on other crops such as *Jatropha* [23].

## Conclusion

In conclusion, the results of this research suggested that a large part of the north and west of Iran is suitable for the cultivation of walnuts. More than 582844 km^2^ (35.36%) of Iran’s total area are currently suitable for walnut cultivation. However, this study considered some parameters only, whereas several other restrictions exist which deserve further inquiry. These include the edaphic pH, along with the availability of water and the soil depth which could undermine the suitability of lands for walnut cultivation. It is an imperative to evaluate the micro-climate of each region and conduct observations in the field before establishing new orchards in each region. In general, the land suitability of walnut cultivation will decrease by 6.20% and reach 546710 km^2^ during the next 30 years (2020–2049). Nonetheless, climate change will affect other environmental parameters including the availability of water, relative humidity, as well as the EC of the water and soil. Since climate change affects these factors too, the unpredictability of an uncertain future for walnuts could be even bleaker than the descriptions made in this research.

## Supporting information

S1 Dataset The meterological data of Iran and delta T_max_ and T_min_ used to prepare walnut land suitability maps for present and future condition. (RAR)

S1 Fig **Classification of Iran’s land for walnut cultivation in the present condition** (in aspect of latitude (a), relative humidity (b), average temperature (c), chilling requirement (e), land slope (e) and soil EC (f) (the map (c) just shows the average temperature in growth season in Iran)).
(DOC)

S2 Fig **Classification of Iran’s land for walnut cultivation in the future (2020–2049)** (regarding maximum (a) and minimum (b) temperature in different months of the year).
(DOC)

S3 Fig The areas of walnut cultivation in Iran based on statistics of the year 2005 which were provided by the Iranian Ministry of Agriculture; each black spot spans about 20 hectares [36]. (DOC)
