# Supplementary material for: Applying the AOGCM-AR5 models to the assessments of land suitability for walnut cultivation in response to climate change: A case study of Iran
Source: PLoS One. 2019 Jun 27;14(6):e0218725. doi: 10.1371/journal.pone.0218725 (PMC6597063; doi:10.1371/journal.pone.0218725)
Supplement: S1 Fig — Classification of Iran’s land for walnut cultivation in the present condition (in aspect of latitude (a), relative humidity (b), average temperature (c), chilling requirement (e), land slope (e) and soil EC (f) (the map (c) just shows the average temperature in growth season in Iran)). (DOC) [file pone.0218725.s002.doc]

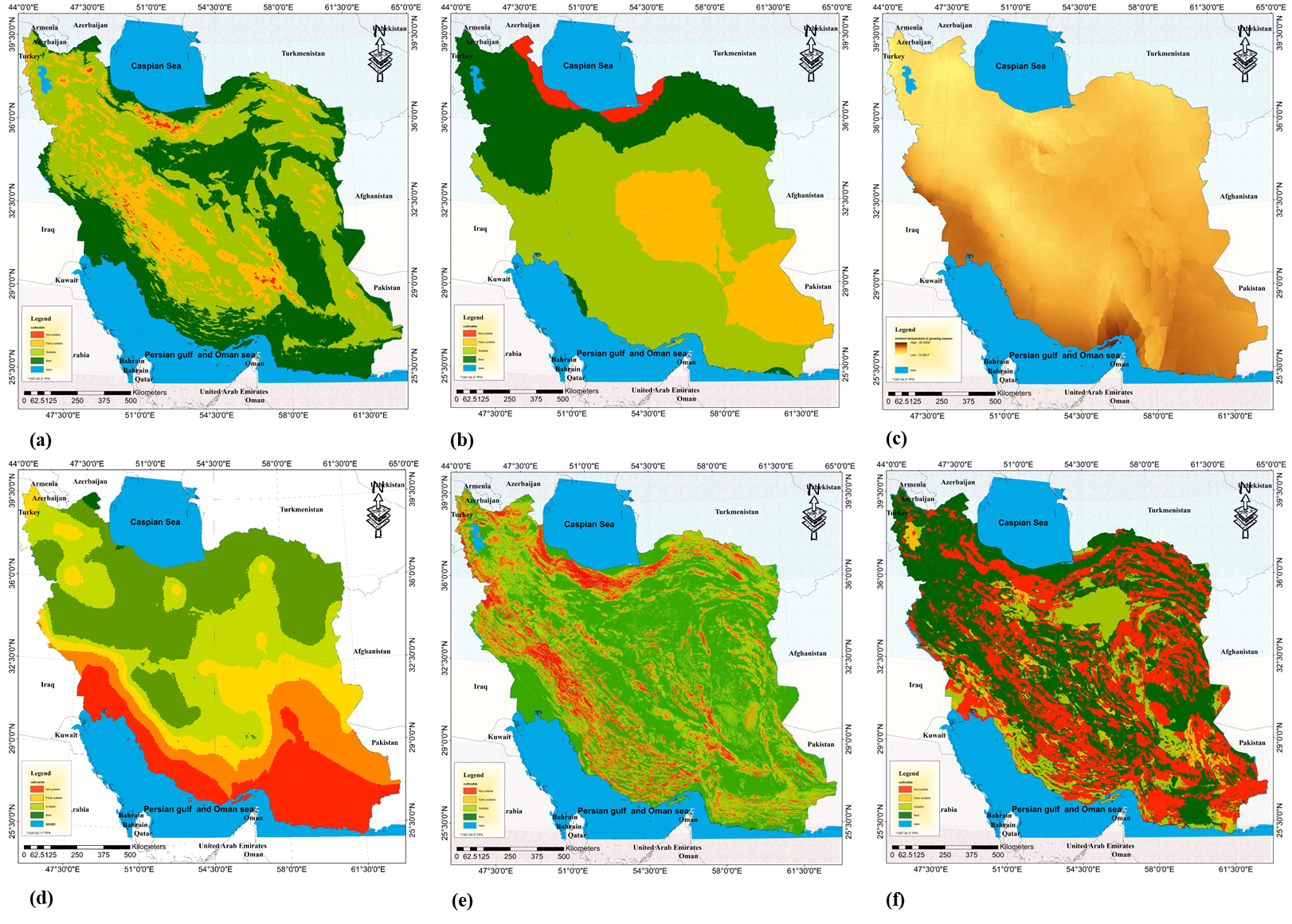


**S1 Fig.** **Classification of Iran for walnut cultivation in the present condition** (in aspect of latitude (a), relative humidity (b), average temperature (c), chilling requirement (e), land slope (e) and soil EC (f) (the map (c) just shows the average temperature in growth season in Iran))
