# Supplementary material for: Applying the AOGCM-AR5 models to the assessments of land suitability for walnut cultivation in response to climate change: A case study of Iran
Source: PLoS One. 2019 Jun 27;14(6):e0218725. doi: 10.1371/journal.pone.0218725 (PMC6597063; doi:10.1371/journal.pone.0218725)
Supplement: S2 Fig — Classification of Iran’s land for walnut cultivation in the future (2020–2049) (regarding maximum (a) and minimum (b) temperature in different months of the year). (DOC) [file pone.0218725.s003.doc]

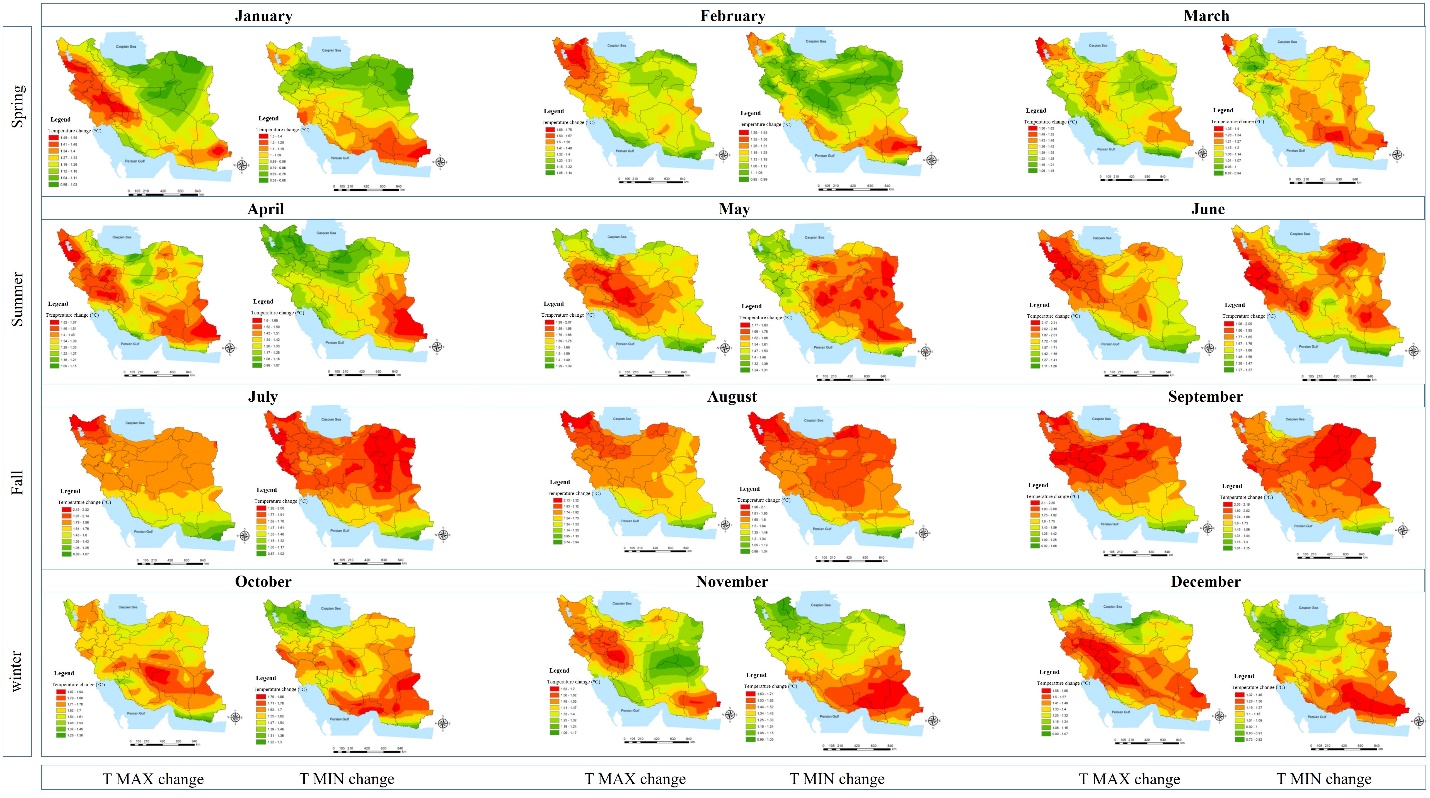


**S2 Fig.** **Classification of Iran for walnut cultivation in the future** (2020-2049) (regarding maximum (a) and minimum (b) temperature in different months of the year)
