# Supplementary material for: Applying the AOGCM-AR5 models to the assessments of land suitability for walnut cultivation in response to climate change: A case study of Iran
Source: PLoS One. 2019 Jun 27;14(6):e0218725. doi: 10.1371/journal.pone.0218725 (PMC6597063; doi:10.1371/journal.pone.0218725)
Supplement: S3 Fig — (DOC) [file pone.0218725.s004.doc]

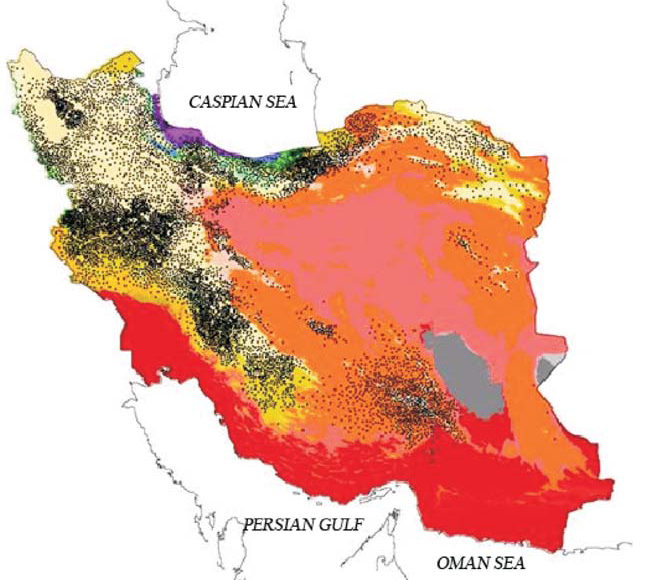


**S3 Fig. The areas of walnut cultivation in Iran based on statistics of the year 2005 which were provided by the Iranian Ministry of Agriculture**; each black spot is about 20 hectares [36]
